# Optimal number of images and 2-year interval affect cancer detection in screening esophagogastroduodenoscopy: An observational study

**DOI:** 10.1097/MD.0000000000038774

**Published:** 2024-06-28

**Authors:** Kazuhiro Kashiwagi, Toshifumi Yoshida, Kayoko Fukuhara, Rieko Bessho, Hitoshi Ichikawa, Nagamu Inoue, Hiromasa Takaishi, Yasushi Iwao, Takanori Kanai

**Affiliations:** a Center for Preventive Medicine, Keio University, Tokyo, Japan; b Hills Joint Research Laboratory for Future Preventive Medicine and Wellness, School of Medicine, Keio University, Tokyo, Japan; c Division of Gastroenterology and Hepatology, Department of Internal Medicine, School of Medicine, Keio University, Tokyo, Japan.

**Keywords:** examination time, number of endoscopic images, screening esophagogastroduodenoscopy, upper gastrointestinal cancer

## Abstract

We aimed to identify quality indicator for esophagogastroduodenoscopy for detecting upper gastrointestinal (UGI) cancer. Data from 43,526 consecutive health checkups from August 2012 to January 2022 were retrospectively collected. The study ultimately analyzed 42,387 examinations by 12 endoscopists who performed more than 1000 examinations, including all cancers detected. These endoscopists were classified either into fast/slow group based on their mean examination time for a normal finding of esophagogastroduodenoscopy during their first year of the examination, or small/large group based on number of endoscopic images, respectively. The association between UGI cancer detection rate and examination time or the number of images was analyzed, using 5 minutes or 50 images as cutoff values. The detection rate of overall (8 pharyngeal, 39 esophageal, 69 gastric) cancers in the fast, slow, small, and large groups were 0.17%, 0.32%, 0.21%, and 0.31%, respectively. On multivariable analysis, endoscopists in the fast group or the small group were less likely to detect overall UGI cancer (OR: 0.596, 95% CI: 0.373–0.952, *P* = .030; OR: 0.652, 95% CI: 0.434–0.979, *P* = .039). Additionally, repeated endoscopy within 2 years had a higher overall cancer detection rate, compared with repeated screening after 2 years. In a sub-analysis, a significant negative relationship was found between the detection rate of gastric cancer and the number of gastric images < 35 (OR: 0.305, 95% CI: 0.189–0.492, *P* = .000). There was also a negative correlation trend between the detection rate of pharyngeal and esophageal cancers and the number of esophageal images < 11 (OR: 0.395, 95% CI: 0.156–1.001, *P* = .050). The optimal number of images and screening 2-year interval are considered useful quality indicators for detecting UGI cancer. This study also suggests that a total of 50 images, or 35 images of the stomach are suitable for detecting UGI cancer, or gastric cancer, during screening endoscopy.

## 1. Introduction

Esophagogastroduodenoscopy (EGD) is widely performed for early detection of cancers as well as nonlesions in the upper gastrointestinal (UGI) tract. The Korean national cancer screening program demonstrated the effect of endoscopic screening on gastric cancer mortality.^[1]^ However, the missing rate for UGI cancers on routine EGD has been reported to vary between 9% and 20%.^[2–6]^ Moreover, according to a Japanese large-scale survey^[7]^ complied 46,529 patients with esophageal cancer experienced at 409 facilities, only 538 (1.2%) patients were detected through health screening program. Therefore, quality indicator for EGD is essential for detecting early UGI cancer in asymptomatic examinees.

Although several guidelines and position statements^[8–10]^ have been published regarding the quality of EGD, no definite quality standards have been established, except for accurate photo-documentation^[11]^ and minimum 7-minute procedure time for first diagnostic UGI endoscopy.^[12]^ In addition, no previous study has investigated the clinical significance of examination time or photo-documentation in the detection of esophageal cancer, except for esophageal adenocarcinoma in Barrett esophagus.^[13,14]^

We hypothesized that endoscopists who perform high-quality endoscopic observations may require longer examination times even in cases with normal EGD findings. Similarly, we thought that they may take more images even if EGD findings are normal. In other words, the endoscopist’s attributes such as examination time and number of endoscopic images may be related to the endoscopic diagnosis rate of UGI cancer. Thus, this study aimed to investigate the association between the detection rate of UGI cancer including esophageal cancer and examination time, or the number of images taken during endoscopic procedure.

## 2. Methods

### 2.1. Subjects and endoscopic procedure

From August 1, 2012 to January 31, 2022, all subjects who underwent UGI endoscopy for a comprehensive health checkup at the center for preventive medicine of our hospital were included for this cross-sectional study. Japanese medical checkup is a more detailed health checkup that includes many test items aimed at early detection of diseases including cancer. Solemio ENDO (Olympus Co., Tokyo, Japan) was used as an endoscopic database. All endoscopic examinations with multiple visits were included in the analyses, and examination experience and intervals from previous examinations in years were calculated. The endoscope system used was Olympus EVIS LUCERA (CLV-260) until July 2019, and Fujifilm LASEREO7000 thereafter and image-enhanced endoscopy (IEE) (narrow band imaging (NBI), linked color imaging, or blue light imaging (BLI)) were available.^[15]^ The main types of endoscopes used were the normal-diameter GIF-Q260 and e.g. L600WR7GIF, or the small diameter GIF-PQ260 and e.g. L580NW7, and neither of them were equipped for non-magnifying observation. Most routes of the insertion were oral, with <0.1% being nasal. As a premedication, pronase MS and sodium bicarbonate each dissolved in dimethicone water was administered, and a viscous method was used in which 5 mL of 2% lidocaine hydrochloride viscous was pooled in the pharynx for 5 minutes for infiltration anesthesia. During the last year, the endoscopists changed a spray method to use 8% lidocaine spray directed into the pharynx. The use of sedatives and analgesics was based on the subject’s wishes, but the final decision was made by each endoscopist. Flunitrazepam or midazolam was used as a sedative, pethidine hydrochloride was used as an analgesic, and past records were referred to for combination and doses of sedatives and analgesics. The use of antispasmodics was also at the discretion of the endoscopist, and scopolamine butyl bromide or glucagon was administered intravenously.

There is no standard manual for endoscopic observation of the pharyngeal and laryngeal region, but images were taken in white light image or image-enhanced mode, during insertion and withdrawal of the scope. The esophagus was observed mainly under white light image during insertion and image-enhanced mode during withdrawal. For subjects diagnosed with esophageal cancer or high risk of esophageal cancer, iodine staining (–1% Lugol solution) of the esophagus was performed, if necessary, after observation with NBI or BLI mode. All endoscopists have at least 5 years of experience and were grouped by whether they were specialists certified by the Japan Gastroenterological Endoscopy Society.

This retrospective research was approved by the Ethics Committee of Keio University School of Medicine (Approval No. 20221001) and was conducted in compliance with the Personal Information Protection Law as an opt-out.

### 2.2. EGD examination time and number of images taken by each endoscopist

Examination time was defined as the time between the first image in the oral cavity captured at scope insertion and the last image at scope withdrawal. The endoscopists were divided into fast and slow examiners, based on the average examination time for normal findings without biopsy nor detailed observation during their first year of working at our center. Also, using this normal finding examination, the average number of total images and the average number of images by each site (oral cavity, esophagus, stomach, and duodenum) taken by each endoscopist were calculated.

### 2.3. Statistical methods

Pharyngeal cancer and esophageal cancer are more common in older men, share common carcinogens such as smoking and alcohol drinking, and often coexist with each other due to the theory of field cancerization.^[16]^ Therefore, we conducted a statistical analysis of pharyngeal cancer and esophageal cancer together. According to the screening intervals of endoscopy prior to the UGI cancer detection, we first classified the examinations into 4 groups: none, ≤1 year, 1–2 years, and >2 years. Then, previous endoscopy was divided into 2 groups (≤2 years, or >2 years), combining ≤1 year and 1 to 2 years groups, or none and >2 years groups, respectively.

For statistical analysis, mean and standard deviation value were described for continuous variables. A *t* test was performed for comparison of the mean values, and a chi-square test was performed for comparison of categorical variables. The relationship between variables was assessed using Pearson’ correlation coefficient or Spearman rank- correlation coefficient, if appropriate. To identify independent predictors of UGI cancer detection, multivariable logistic regression analysis was performed, including age, sex, previous endoscopy, sedation, normal-diameter endoscopy, endoscope system, specialist qualified, examination time or number of endoscopic images as independent variables. SPSS ver24 was used for the analysis, and the significance level was judged to be significant when *P* < .05.

## 3. Results

### 3.1. Characteristics of participants

**Figure 1.**
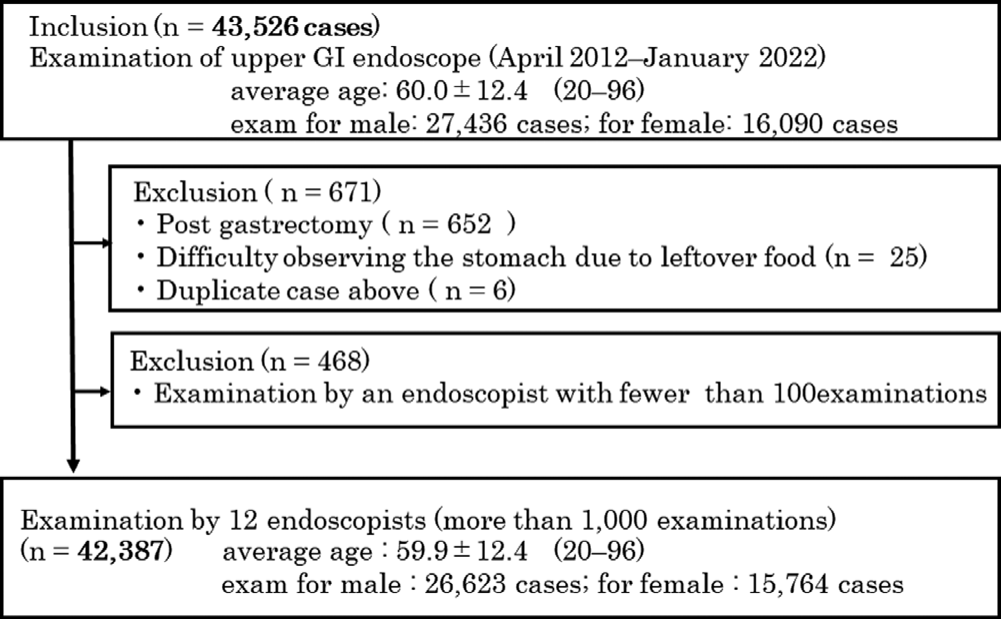
Flow chart of the present study.

Of 43,526 consecutive health checkups, 42,387 (97.4%) performed by 12 endoscopists who examined more than 1000 EGDs were finally analyzed (Fig. 1). These cases included 26,623 (62.8%) males, with a mean age of 59.9 ± 12.4 years (Table 1). There were 1185 (2.8%) normal EGD examinations, with mean examination time of 337 (range, 242–470) seconds and mean number of total images of 50.4 (33–68.3) (Table 2). Table 1 shows the characteristics of subjects divided into 2 endoscopist groups. Based on previous reports,^[12,17,18]^ 3 endoscopists were classified into the fast and 9 into the slow groups, using a cutoff value of 5 minutes for the examination time. Also, 7 were classified into the small and 5 into the large groups, using a cutoff value of 50 for the total images taken, since there were no reports regarding the optimal number of images and the overall average number of images was 50.4. A total of 31% and 38% belonged to the fast and the small group, respectively. Most of the subjects underwent EGD under sedation (94.7%) by board-certified endoscopists (88.9%).

**Table 1:** Clinical characteristics in the short and long examination time groups, or the small and large number of images taken groups.

Characteristics of subjects	Total	Examination time < 5 min (fast)	Examination time ≥ 5 min (slow)	*P*	Number of images < 50 (small)	Number of images ≥ 50 (large)	*P*
N (%)	42,387	12,908 (31)	29,479 (69)		16,104 (38)	26,283 (62)	
Male	26,623 (62.8)	7923 (61.4)	18,700 (63.4)	**.000**	10,157 (63.1)	16,466 (62.6)	.382
Age, yr (SD)	59.9 (12.4)	59.9 (12.4)	59.9 (12.5)	.784	60.3 (12.4)	59.6 (12.4)	**.000**
Sedation	40,128 (94.7)	12,187 (94.4)	27,941 (94.8)	.120	15,201 (94.4)	24,927 (94.8)	**.046**
Normal-diameter endoscope, N (%)	28,633 (67.6)	8596 (66.6)	20,037 (68.0)	**.005**	9894 (61.4)	18,739 (71.3)	**.000**
Endoscope system (Olympus), N (%)	36,943 (87.2)	11,191 (86.7)	25,752 (87.4)	.062	13,975 (86.8)	22,968 (87.4)	.070
Previous endoscopy, N (%)							
None	13,545 (32.0)	3177 (24.6)	10,368 (35.2)	**.000**	4727 (29.4)	8818 (33.6)	**.000**
≤1 yr	23,646 (55.7)	7868 (61.0)	15,778 (53.5)		9319 (57.9)	14,327 (54.5)	
1 to 2 yr	3888 (9.2)	1385 (10.7)	2503 (8.5)		1536 (9.5)	2352 (8.9)	
>2 yr	1308 (3.1)	478 (3.7)	830 (2.8)		522 (3.2)	786 (3.0)	
Specialist qualified, N (%)	37,684 (88.9)	10,679 (82.7)	27,005 (91.6)	**.000**	12,659 (78.6)	25,025 (95.2)	**.000**
UGI cancer, N (%)	* 116 (0.274)	22 (0.170)	94 (0.319)	.010	34 (0.211)	82 (0.312)	.046
Pharyngeal cancer, N (%)	8 (0.019)	2 (0.015)	6 (0.020)	.540	2 (0.012)	6 (0.023)	.369
Esophageal cancer, N (%)	39 (0.092)	11 (0.085)	28 (0.095)	.760	13 (0.081)	26 (0.099)	.549
Gastric cancer, N (%)	69 (0.163)	9 (0.070)	60 (0.204)	.002	19 (0.118)	50 (0.190)	.073
Duodenal cancer, N (%)	0	0	0		0	0	

SD = standard deviation, UGI = upper gastrointestinal.

* Including 1 patent each with pharyngeal and esophageal cancer, and 1 patient with esophageal and gastric cancer.

**Table 2:** Detection rate of total cancers, examination time, number of images and biopsy rate by each endoscopist.

Endoscopist	Detection rate of total cancers (%)	Examination time (s)	Total number of images	Number of images of the esophagus	Number of images of the stomach	Biopsy rate (%)
	0.26 (0.09–0.63)	337 (242–470)	50.4 (33–68.3)	12.4 (5.5–20)	30.0 (18.8–38.4)	4.63 (0.27–16.22)
A	0.25	324	49	10.5	29.4	3.29
B	0.16	309	54.5	10.9	37.3	16.22
C	0.08	448	48.7	10.4	32.2	1.47
D	0.42	371	44.6	14.6	22.2	5.77
E	0.22	339	47.4	12.6	26.9	6.88
F	0.63	470	68.3	20	37.2	9.17
G	0.09	242	33	5.5	18.8	0.27
H	0.18	305	45.2	10.7	27.7	2.62
I	0.26	256	47.2	12.2	28.6	1.97
J	0.16	252	50.5	11.9	30.2	1.14
K	0.16	384	58.5	17.6	31.6	2.12
L	0.51	347	57.8	11.5	38.4	4.65

### 3.2. UGI cancer detection rate and its association with examination time or number of images taken by 12 endoscopists

As shown in Table 1, a total of 116 UGI cancers were detected in 114 participants (0.274%), of whom 8 had pharynx cancers, 39 had esophageal cancers, and 69 had gastric cancers. The detection rate of overall cancer in the fast, slow, small, and large groups were 0.170%, 0.319%, 0.211%, and 0.312%, respectively. The detection rates of overall cancers per endoscopist ranged from 0.08% to 0.63% (Table 2).

**Figure 2.**
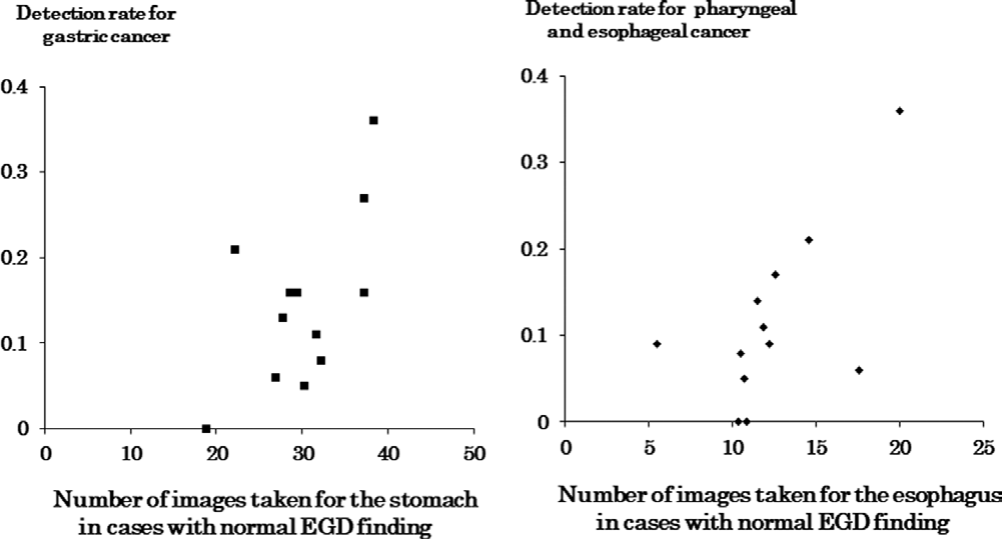
Correlation coefficient between cancer detection rate and number of endoscopic images. (Lt) Detection rate for gastric cancer and number of endoscopic images taken for the stomach in cases with normal EGD finding. (Rt) Detection rate for pharyngeal and esophageal cancer, and number of endoscopic images taken for the esophagus in cases with normal EGD finding. EGD = esophagogastroduodenoscopy.

In Figure 2, we investigated the correlation coefficient between the detection rate of gastric cancer, or pharyngeal and esophageal cancer, and the number of images taken for the stomach, or the esophagus, in cases with normal EGD finding, respectively. The number of images for each site was significantly correlated with the detection rate of gastric cancer (*R* = 0.608, *P* = .036), or pharyngeal and esophageal cancer (*R* = 0.624, *P* = .030).

### 3.3. Factors associated with the detection of UGI cancer

Due to the high multicollinearity of examination time and the number of images taken, they were separately included in the multivariable regression model when analyzing the factors involved in overall UGI cancer detection. As shown in Table 3, overall UGI cancer detection rates were significantly higher among subjects who were male, older, and who had not undergone an endoscopy within the past 2 years (previous endoscopy (>2 years)). Moreover, endoscopist with fast examination time or with the small number of total images were not more likely to detect overall UGI cancer (OR: 0.596, 95% CI: 0.373–0.952, *P* = .030; OR: 0.652, 95% CI: 0.434–0.979, *P* = .039).

**Table 3:** Factors associated with detection of a histologically verified upper gastrointestinal cancer.

Variables	Multivariate analysis 1			Multivariate analysis 2		
	OR	95% CI	*P*	OR	95% CI	*P*
Examinee’s factors						
Male	6.890	3.484 to 13.267	**.000**	6.983	3.531 to 13.811	**.000**
Age	1.064	1.046 to 1.082	**.000**	1.065	1.047 to 1.083	**.000**
Previous endoscopy (>2 yr)	1.706	1.168 to 2.493	**.006**	1.761	1.207 to 2.568	**.003**
Endoscopist’s factors						
Examination time (<5 min)	0.596	0.373 to 0.952	**.030**	N/A		
Number of images (<50)	N/A			0.652	0.434 to 0.979	**.039**
Specialist qualified			.074			.093
Other factors						
Sedation			.058			.056
Normal-diameter endoscope			.897			.988
Endoscope system			.372			.383

CI = confidence interval, N/A = not available, OR = odds ratio.

We further analyzed the effect of number of images on cancer detection for the pharynx and esophagus, or stomach, respectively. In the sub-analysis of Table 4, there was a significant relationship between the gastric cancer detection rate and the number of gastric images < 35 (OR: 0.305, 95% CI: 0.189–0.492, *P* = .000). There was also a trend between the detection rate of pharyngeal and esophageal cancer and the number of esophageal images < 11 (OR: 0.395, 95% CI: 0.156–1.001, *P* = .050). In addition, gastric cancer detection was significantly correlated with previous endoscopy (>2 years) (OR: 1.682, 95% CI: 1.037–2.730, *P* = .035), although the association between the detection rate of pharyngeal and esophageal cancers and previous endoscopy (>2 years) did not reach a statistical significance (*P* = .055).

**Table 4:** Factors associated with detection of gastric cancer and esophageal cancer.

Variables	Gastric cancer			Pharyngeal and esophageal cancer		
	OR	95% CI	*P*	OR	95% CI	*P*
Male	7.737	3.110 to 19.250	**.000**	6.000	2.151 to 16.739	**.001**
Age	1.065	1.043 to 1.088	**.000**	1.051	1.025 to 1.078	**.000**
Number of gastric images < 35	0.305	0.189 to 0.492	**.000**	N/A		
Number of esophageal images < 11	N/A			0.395	0.156 to 1.001	.050
Previous endoscopy (>2 yr)	1.682	1.037 to 2.730	**.035**			.055
Sedation			.054			.367
Normal-diameter endoscope			.331			.182
Endoscope system			.177			.647
Specialist qualified			.069			.612

CI = confidence interval, N/A = not available, OR = odds ratio.

## 4. Discussion

The best quality indicator for EGD would be to measure the incidence of cancer or death after endoscopic examination. To increase the quality of EGD examination, several guidelines and statements^[8–10]^ recommend performance measures for UGI endoscopy. In this study, age and male sex, as well as the duration of EGD examination were found to be associated with the detection of a histologically confirmed overall UGI cancer. We observed the positive correlation between the detection rate of UGI cancer and EGD examination time or the number of images by using 5 minutes or 50 images as cutoffs, respectively. The former finding is in line with previously published data mainly focused on gastric cancer and/or gastric precancerous lesions.^[8,9]^

Teh et al^[12]^ defined procedure time as the time from inserting the endoscope into the patient’s mouth until withdrawing it, while Park et al^[17]^ defined observation time as the time from when the endoscope reached into the duodenum until it was withdrawn. Since their procedure time and observation time was 7 minutes and 3 minutes, respectively, our examination time of 5 minutes seems to be closer to that of the latter group. Moreover, Kawamura et al^[18]^ concluded that the odds ratio for the detection rate of neoplastic lesion in asymptomatic examinees in the moderate and slow groups were 1.90 and 1.89, compared with that in the fast group, by using cutoff times of 5 and 7 minutes. Our results were almost similar to those from the cross-sectional study of the Japanese group, demonstrating the validity of the 5-minute cutoff.

Our study highlights that the number of endoscopic images is also a useful quality indicator for screening EGD, because endoscopists who took a small number of images (<50) were not likely to detect overall UGI cancer. Photo-documentation of anatomical landmarks is regarded as a proof of a complete EGD procedure. The minimum requirements for photo-documentation vary worldwide. The European Society of Gastrointestinal Endoscopy (ESGE) recommends taking images of a minimum of 8^[11]^ to 10^[8]^ anatomical landmarks. On the other hand, the Japanese systematic screening protocol for the stomach proposed 22 images of the stomach as a minimum required standard to avoid blind spots.^[19]^ Wang et al^[20]^ concluded that the standardized white light gastroscopy, which obtained at least 34 images, including 5 images for the esophagus and 22 images of the stomach, contributed to improve the detection rate of gastric lesions. In our endoscopy, the average number of images for the esophagus or stomach was approximately 12 or 30, respectively, and bivariate correlation analysis suggested that 11 esophageal images, or 35 gastric images are suitable for detecting pharyngeal and esophageal cancer, or gastric cancer, respectively, in screening EGD. Obtaining and observing still images in addition to video observation may have an additional impact on the detection of cancer. Moreover, the ampulla photo-documentation rate, which was closely related with observation time for a normal EGD, significantly correlated with the detection rate for both total and small UGI neoplasms for asymptomatic patients.^[21]^ Romańczyk et al^[22]^ reported that the detection of UGI neoplasms for symptomatic patients was associated with composite detection rate, based on the detection at least 1 of 3 mostly benign lesions: esophageal inlet patch, gastric polyp and post-ulcer duodenal bulb deformation. However, most of the neoplasms detected by both groups were in the stomach. Although photo-documentation of these checkpoints may ensure a meticulous examination, it remains to be demonstrated whether this may have an additional impact on the detection of UGI cancer other than gastric cancer.

In our cohort, the frequency of endoscopic biopsies varied significantly between endoscopists (mean, 4.6%; range, 0.3–16.2%) (Table 2). A linear relationship was not observed in the endoscopist biopsy rate and the cancer detection rate (*R* = 0.558, *P* = .059) for asymptomatic subjects. Januszewicz et al^[23]^ reported that the endoscopist biopsy rate (mean, 43.8%; range, 22.4–65.8%) was associated with efficacy in detecting gastric premalignant conditions and the rate of missed gastric cancers for outpatients with GI symptoms. This difference in biopsy rates may be due in part to the presence or absence of GI symptom for the subjects. For the appropriate endoscopic screening for gastric cancer in Japan, where the prevalence of chronic gastritis and gastric cancer is high, the biopsy rate is recommended to be kept within 10% to avoid complications such as bleeding as much as possible.^[24]^ Moreover, an Asian consensus on standards of diagnostic upper endoscopy for neoplasia^[25]^ includes some guidance. For example, the use of IEE in addition to white light image improves the detection rate for esophageal superficial neoplasm, and the use of equipment-based IEE without magnification characterizes superficial esophageal neoplasm and early gastric neoplasm. “Optical biopsy” using such advanced imaging technique is popular in Japan, since the use of IEE techniques would prolong examination time as well as increase the detection of neoplastic lesions, it may be possible confounder. However, all of us endoscopists use these IEE techniques to detect and observe suspicious UGI malignancies and also use NBI or BLI when withdrawing endoscope during the observation of the esophagus in daily clinical practice. Therefore, the use of imaging technology appears to have a negligible impact on the tumor detection rate and examination time between our endoscopists. “Optical biopsy” may also explain the low biopsy rate during our examination.

As for the optimal interval between endoscopies, we believe that repeated screening endoscopy within 2 years may be more effective for the detection of overall UGI cancer, including gastric cancer. Reports from the Korean and Japan that recommended biannual upper endoscopy for adults aged ≥40 or ≥50, respectively,^[26,27]^ are consistent with our results. On the other hand, according to British Society of Gastroenterology and the Management of Epithelial Precancerous Conditions and Lesions in the Stomach, endoscopic surveillance is recommended every 3 years for individuals with severe atrophy or intestinal metaplasia, and within 1 year for low grade intraepithelial neoplasia.^[28,29]^ Future large-scale studies are essential to validate the appropriate time interval for repeated screening depending on the severity of gastric lesions.

Our study has several strong points. This is the first study to investigate the impact of the number of images for screening EGD. The study included all consecutive cases in a cohort, analyzed histologically confirmed UGI cancers, including 39 esophageal cancers. We believe we could control many confounders affecting cancer detection through endoscopic procedures. In fact, mucolytic and defoaming agents were administered to all subjects and most subjects (94.7%) were given sedatives, according to the statement for improving the cancer detection rate.^[20]^ However, the present study has some limitations. First, the present results were taken from 12 endoscopists at a single center having large volumes of screening UGI endoscopies. These results may have a limitation of generalizability beyond this environment. Second, we could not examine other risk factors for UGI cancers such as patient factors (i.e., smoking, alcohol, family history, and Helicobacter infection). Third, we were not able to demonstrate an appropriate number of images for the esophagus to improve the detection rate of pharyngeal and esophageal cancer. Finally, we did not evaluate missed cases in the present study. However, the possibility of false-negative examination on EGD procedure is considered to be very low, since most of the participants (68.2%) underwent EGD repeatedly at our center.

In conclusion, our retrospective analysis demonstrates that not only examination time but also optimal number of images are considered useful quality indicators for detecting UGI cancers. Furthermore, it was suggested that repeated screening endoscopy within 2 years may be more effective. This study also suggests that a total of 50 images, or 35 images of the stomach are suitable for detecting UGI cancer, or gastric cancer, during endoscopy in non-symptomatic subjects. Our results require further validation study using these indicators to determine whether they are correlated with improved early UGI cancer detection and reduced cancer missed rate.

## Acknowledgments

The authors would like to thank the endoscopists and the medical staff of the Endoscopy Division at Center for Preventive Medicine, Keio University. KK is a member of Hills Joint Research Laboratory for Future Preventive Medicine and Wellness funded by Mori Building Co., Ltd (https://www.mori.co.jp). The funders had no role in study design, data collection, and analysis, decision to publish, or preparation of the manuscript.

## Author contributions

**Conceptualization:** Kazuhiro Kashiwagi, Takanori Kanai.

**Investigation:** Kazuhiro Kashiwagi, Toshifumi Yoshida, Kayoko Fukuhara, Rieko Bessho, Hitoshi Ichikawa, Nagamu Inoue, Yasushi Iwao.

**Project administration:** Kazuhiro Kashiwagi, Hiromasa Takaishi, Yasushi Iwao.

**Supervision:** Kazuhiro Kashiwagi, Takanori Kanai.

**Writing – original draft:** Kazuhiro Kashiwagi, Toshifumi Yoshida.

**Writing – review & editing:** Kazuhiro Kashiwagi, Toshifumi Yoshida, Nagamu Inoue, Hiromasa Takaishi, Yasushi Iwao, Takanori Kanai.

**Data curation:** Toshifumi Yoshida.

**Formal analysis:** Toshifumi Yoshida, Hitoshi Ichikawa, Nagamu Inoue.

**Methodology:** Nagamu Inoue.

## Correction

This article was originally published with Kazuhiro Kashiwagi spelled incorrectly as Kazuhiro Ksahiwagi. It has now been corrected in the online version.

## References

[1] Jun JK Choi KS Lee HY. Effectiveness of the Korean National Cancer Screening Program in reducing gastric cancer mortality. Gastroenterology. 2017;152:1319–28.e7. 28147224 10.1053/j.gastro.2017.01.029

[2] Hosokawa O Tsuda S Kidani E. Diagnosis of gastric cancer up to three years after negative upper gastrointestinal endoscopy. Endoscopy. 1998;30:669–74. 9865554 10.1055/s-2007-1001386

[3] Yalamarthi S Witherspoon P McCole D. Missed diagnoses in patients with upper gastrointestinal cancers. Endoscopy. 2004;36:874–9. 15452783 10.1055/s-2004-825853

[4] Pimenta-Melo AR Monteiro-Soares M Libânio D. Missing rate for gastric cancer during upper gastrointestinal endoscopy: a systematic review and meta-analysis. Eur J Gastroenterol Hepatol. 2016;28:1041–9. 27148773 10.1097/MEG.0000000000000657

[5] Alexandre L Tsilegeridis-Legeris T Lam S. Clinical and endoscopic characteristics associated with post-endoscopy upper gastrointestinal cancers: a systematic review and meta-analysis. Gastroenterology. 2022;162:1123–35. 34958760 10.1053/j.gastro.2021.12.270

[6] Kamran U King D Abbasi A. A root cause analysis system to establish the most plausible explanation for post-endoscopy upper gastrointestinal cancer. Endoscopy. 2023;55:109–18. 36044914 10.1055/a-1917-0192

[7] Rikitake R Ando M Saito Y. Current status of superficial pharyngeal squamous cell carcinoma in Japan. Int J Clin Oncol. 2017;22:826–33. 28501947 10.1007/s10147-017-1135-9

[8] Bisschops R Areia M Coron E. Performance measures for upper gastrointestinal endoscopy: a European Society of Gastrointestinal Endoscpy (ESGE) quality improvement initiative. Endoscopy. 2026;48:843–64. 10.1055/s-0042-113128 27548885

[9] Park WG Shaheen NJ Cohen J. Quality indicators for EGD. Am J Gastroenterol. 2015;110:60–71. 25448872 10.1038/ajg.2014.384

[10] Beg S Ragunath K Wyman A. Quality standards in upper gastrointestinal endoscopy: a position statement of the British Society of gastroenterology (BSG) and Association of Upper Gastrointestinal Surgeons of Great Britain and Ireland (AUGIS). Gut. 2017;66:1886–99. 28821598 10.1136/gutjnl-2017-314109 PMC5739858

[11] Rey JF Lambert R; ESGE Quality Assurance Committee. ESGE recommendations for quality control in gastrointestinal endoscopy: guidelines for image documentation in upper and lower GI endoscopy. Endoscopy. 2001;33:901–3. 11605605 10.1055/s-2001-42537

[12] Teh JL Tan JR Lau LJF. Longer examination time improves detection of gastric cancer during diagnostic upper gastrointestinal endoscopy. Clin Gastroenterol Hepatol. 2015;13:480–7.e2. 25117772 10.1016/j.cgh.2014.07.059

[13] Gupta N Gaddam S Wani SB. Longer inspection time is associated with increased detection of high-grade dysplasia and esophageal adenocarcinoma in Barrett’s esophagus. Gastrointest Endosc. 2012;76:531–8. 22732877 10.1016/j.gie.2012.04.470

[14] Kim SY Park JM. Quality indicators in esophagogatroduodenoscopy. Clin Endosc. 2022;55:319–31. 35656624 10.5946/ce.2022.094 PMC9178133

[15] Rodríguez-Carrasco M Esposito G Libânio D. Image-enhanced endoscopy for gastric preneoplastic conditions and neoplastic lesions: a systematic review and meta-analysis. Endoscopy. 2020;52:1048–65. 32663879 10.1055/a-1205-0570

[16] Chung CS Liao LJ Wu CY. Endoscopic screening for second primary tumors of the esophagus among head and neck cancer patients. Front Oncol. 2022;12:906125. 35747824 10.3389/fonc.2022.906125 PMC9209650

[17] Park JM Huo SM Lee HH. Longer observation time increases proportion of neoplasms detected by esophagogastroduodenoscopy. Gastroenterology. 2017;153:460–9.e1. 28501581 10.1053/j.gastro.2017.05.009

[18] Kawamura T Wada H Sakiyama N. Examination time as a quality indicator of screening upper gastrointestinal endoscopy for asymptomatic examinees. Dig Endosc. 2017;29:569–75. 28066945 10.1111/den.12804

[19] Yao K. The endoscopic diagnosis of early gastric cancer. Ann Gastroenterol. 2013;26:11–22. 24714327 PMC3959505

[20] Wang Q Zhang SY Wu X. Feasibility of standardized procedures of white light gastroscopy for clinical practice: a multicenter study in China. J Dig Dis. 2021;22:656–62. 34693636 10.1111/1751-2980.13061

[21] Park JM Lim CH Cho YK. The effect of photo-documentation of the ampulla on neoplasm detection rate during esophagogastroduodenoscopy. Endoscopy. 2019;51:115–24. 30184610 10.1055/a-0662-5523

[22] Romańczyk M Ostrowski B Marek T. Composite detection rate as an upper gastrointestinal endoscopy quality measure correlating with detection of neoplasia. J Gastroenterol. 2021;56:651–8. 33934197 10.1007/s00535-021-01790-3 PMC8280029

[23] Januszewicz W Wieszczy P Bialek A. Endoscopic biopsy rate as a quality indicator for our patient gastroscopy: a multicenter cohort study with validation. Gastrointest Endosc. 2019;89:1141–9. 30659831 10.1016/j.gie.2019.01.008

[24] Hamashima C Fukao A. Quality assurance manual of endoscopic screening for gastric cancer in Japanese communities. Jpn J Clin Oncol. 2016;46:1053–61. 27589938 10.1093/jjco/hyw106

[25] Chiu PWY Uedo N Singh R. An Asian consensus on standards of diagnostic upper endoscopy for neoplasia. Gut. 2019;68:186–97. 30420400 10.1136/gutjnl-2018-317111

[26] Suh M Song S Cho HN. Trends in participation rates for the national cancer screening program in Korea, 2002–2012. Cancer Res Treat. 2017;49:798–806. 27857022 10.4143/crt.2016.186 PMC5512374

[27] Hamashima C. Cancer screening guidelines and policy making: 15 years of experience in cancer screening guideline development in Japan. Jpn J Clin Oncol. 2018;48:278–86. 29315389 10.1093/jjco/hyx190

[28] Banks M Graham D Jansen M. British Society for Gastoenterology guidelines on the diagnosis and management of patients at risk of gastric adenocarcinoma. Gut. 2019;68:1545–75. 31278206 10.1136/gutjnl-2018-318126 PMC6709778

[29] Pimentel-Nunes P Libânio D Marcos-Pinto R. Management of epithelial precancerous conditions and lesions in the stomach (MAPS II): European Society of gastrointestinal Endoscopy (ESGE), European Helicobacter and Microbiota Study Group (EHMSG), European Society of Pathology (ESP), and Sociedade Portuguesa de Endoscopia Digestiva (SPED) guideline update 2019. Endoscopy. 2019;51:365–88. 30841008 10.1055/a-0859-1883

